# A Direct and an Efficient Regioselective Synthesis of 1,2-Benzothiazine 1,1-dioxides, β-Carbolinones, Indolo[2,3-*c*]pyran-1-ones, Indolo[3,2-*c*]pyran-1-ones, Thieno[2,3-*c*]pyran-7-ones and Pyrano[3’,4’:4,5]imidazo[1,2-*a*]pyridin-1-ones via Tandem Stille/Heterocyclization Reaction

**DOI:** 10.3390/molecules25215137

**Published:** 2020-11-04

**Authors:** Badr Jismy, Khalil Cherry, Carine Maaliki, Samuel Inack Ngi, Mohamed Abarbri

**Affiliations:** 1 Laboratoire de Physico-Chimie des Matériaux et des Electrolytes Pour l’Energie (PCM2E), EA 6299, Avenue Monge, Faculté des Sciences, Université de Tours, Parc de Grandmont, 37200 Tours, France; badr.jismy@hotmail.com (B.J.); Carine.mouchaham@univ-tours.fr (C.M.); 2 Laboratoire Matériaux, Catalyse, Environnement et Méthodes Analytiques (MCEMA), Campus Universitaire de Hadat, Université Libanaise, Beyrouth, Lebanon; khalilcherry@hotmail.com; 3 IKAMBA Organics, rue Edouard Branly, 37230 Fondettes, France; Samuel.inack@ikambaorganics.com

**Keywords:** regioselective synthesis, 1,2-benzothiazine 1,1-dioxides, β-carbolinones, indolo[2,3-*c*] and [3,2-*c*]pyrane-1-one derivatives, Stille/Heterocyclization reaction

## Abstract

A general regioselective one-pot synthesis of 1,2-benzothiazine 1,1-dioxides from 2-iodo benzenesulfonamide moieties and allenylstannanes is described using a domino Stille-like/Azacyclization reaction. The conditions developed also opened a novel access to β-carbolinones, indolopyranones, thienopyranones and pyrano-imidazopyridines.

## 1. Introduction

With the development of efficient cross-coupling catalysts, the heterocyclic synthesis of compounds of various interests has been facilitated in terms of numerous parameters such as temperature, catalytic charge, selectivity or efficiency. Several methodologies have been set up to create carbon-carbon or carbon-heteroatom bonds and applied to intramolecular cyclization and heterocyclization reactions. The development of palladium cross-coupling processes in particular has enabled easy access to various cores with oxygen, nitrogen or sulfur as heteroelement.

**Figure 1:**
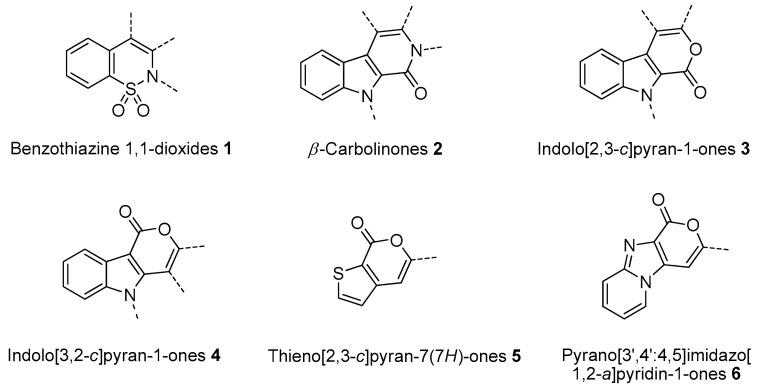
General structures of synthesized cores.

In this paper we focus on 1,2-benzothiazine 1,1-dioxide also known as benzosultam (**1**), β-carbolinone (**2**), pyranoindole (**3**,**4**), thienopyranone (**5**) and pyrano-imidazopyridine (**6)** cores (Figure 1).

**Figure 2:**
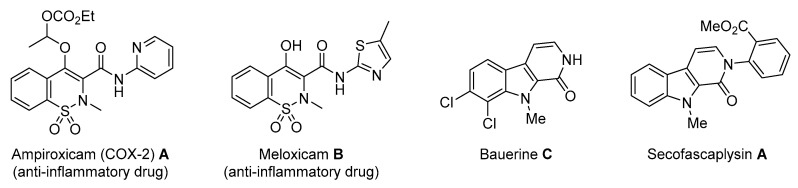
Biologically active benzothiazine dioxides and β-carbolin-1-ones.

Molecules possessing the moiety **3** are known to inhibit the hepatitis C virus NS5B polymerase [1,2] while 1,2-benzothiazine 1,1-dioxide derivatives possess versatile biological activities [3,4]. For example the **1** moiety constitutes the heterocyclic core of oxicam (e.g., Ampiroxicam **A** and meloxicam **B**, Figure 2), a class of non-steroidal anti-inflammatory drugs [3]. In addition, benzothiazine dioxide derivatives exhibit strong inhibitory properties against HIV integrand [5], and Calpain 1 [6]. Compounds **3** and **4** may constitute interesting precursors to β- and γ-carbolinone alkaloids respectively. The β-carbolin-1-ones skeleton **2** is found in the structure of numerous natural products of which some derivatives act on the central nervous system [7,8] and are recognized as HeLa and HLE cancer cell line inhibitors [9]. β-carbolin-1-ones are also important intermediates for the synthesis of many alkaloids [10,11], such as Bauerine C [12] and Secofascaplysin A [13] (Figure 2), and they are effective as anti-diabetics ATAD25 agents [14] and inhibitors [15]. Pyrano [3’,4’:4,5]imidazo[1,2-*a*]pyridin-1-one derivatives are known for their interesting pharmacological activities particularly as antitumoral agents [16].

**Scheme 1:**
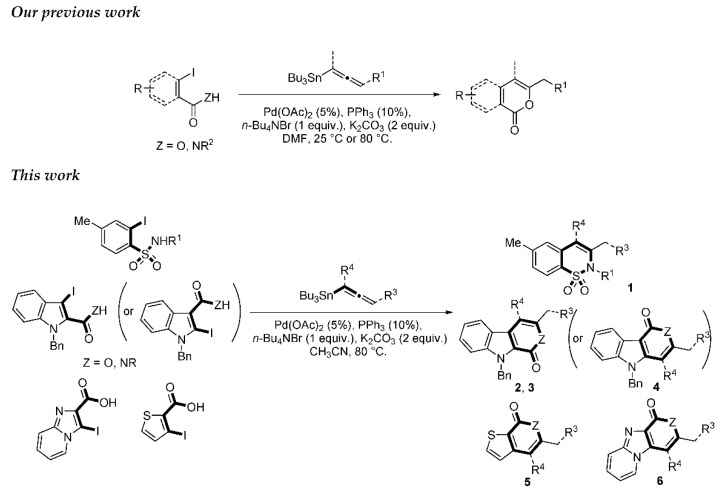
Convergent Stille coupling/heterocyclization reaction of β-iodo-α,β-unsaturated carboxylic acid or carboxamide systems.

Numerous ways to access these moieties are depicted in the literature. The most popular route used is metal catalyzed bond formation [17,18,19,20,21,22,23,24]. For example, β-carbolinones are accessed via Heck [15], gold cycloisomerization [25] or copper catalyzed C-N bond formation [26]. Other synthetic approaches for β-carbolinones have been developed via phase-transfer catalyzed intramolecular cyclization of 3-alkynylindole-2-carboxamides [27], intramolecular Diels-Alder reaction [28] and cyclization of pyridone ring [8,29]. Recently, Xia et al. described the synthesis of β-carbolinones via Pd/Cu catalyzed tandem C-H aminocarbonylation and dehydrogenation of tryptamines [30]. Although the 1,2-benzothiazine 1,1-dioxide core is widely accessed via Heck coupling [31], other metal catalyzed methods like domino Sonogashira-azacyclization [18], silver assisted aza-cyclization of enyne [19], gold-catalyzed cycloisomerization of terminal alkene [32], gold(I)-catalyzed ammonium formation strategy [33], Rh(III)-ctalyzed strategy by the *ortho* C-H activation [34] or C-H bond alkynylation of aryl sulfonamides [21] have been published. Moreover, 1,2-benzothiazine 1,1-dioxides has been achieved by a palladium-catalyzed tandem-cyclization of ynamides [35]. Recently, Volla et al. [36] reported a cobalt-catalyzed C-H activation of arylsulfonamides and their intermolecular heteroannulation reaction with allenes for the synthesis in a highly regioselective manner of aryl fused sultams. Due to their various pharmacological importances, the development of a novel and simple synthetic method for the synthesis of benzothiazine dioxide and derivatives would be highly desirable. Metal-catalyzed transition carbon-carbon bond formation has attracted much attention over the last three decades. The palladium-catalyzed cross-coupling reaction is one of the most efficient methods for the construction of C-C bonds. These reactions are frequently employed to promote the synthesis of numerous natural products or bio-active molecules. Among them the value of the Stille cross-coupling reaction [37] is commonly recognized in the scientific community and the reactivity of aromatic halide is widely known using that methodology. We previously published an easy and mild palladium-catalyzed process for rapid access to α-pyrones, α-pyridones and isocoumarins by one-pot approaches involving the intramolecular addition of carboxylic acid derivatives to allenyl moiety (Scheme 1). The reactions proceeded as a tandem coupling heterocyclization sequence in the presence of palladium catalyst and an alkaline carbonate.

We report here a valuable synthetic extension of this method onto aromatic and heteroaromatic substrates such as *o*-iodo arylsulfonamide, indole, thiophene and imidazo[1,2-*a*]pyridine bearing a β-iodo-α,β-unsaturated carboxylic acid or carboxamide system in order to produce **1**, **2**, **3**, **4, 5** and **6** cores (Scheme 1). To the best of our knowledge, no cross-coupling using allenyltin reagent has been reported to date to access to these compounds.

## 2. Results and Discussion

**Scheme 2:**
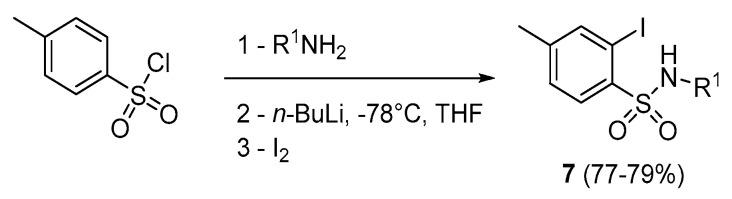
Synthesis of 2-iodo benzenesulfonamide derivatives.

Our investigations began with assays on aromatic sulfonamides (**7**) for the synthesis of 1,2-benzothiazine 1,1-dioxide derivatives **1**. The required **7** were prepared from the corresponding sulfonyl chloride by treatment with alkylamines, followed by a reaction with *n*-BuLi then elemental iodine according to the procedure reported in the literature (Scheme 2)[38,39].

**Scheme 3:**
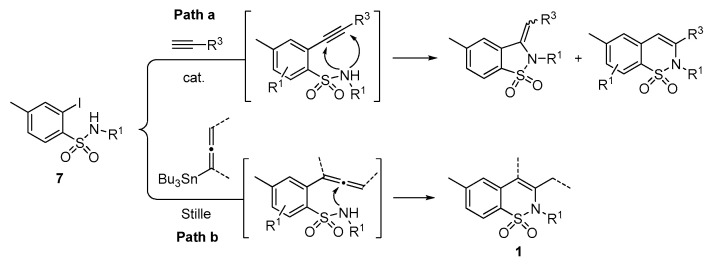
Difference between alkyne and allenyltin reagent in the synthetic paths of 2*H*-1,2-benzothiazine 1,1-dioxide derivatives.

A series of experiments on the same basis as in our previous work was carried out to optimise the reaction conditions and establish the minimum requirements for this process. It was found that for good performance one needs at least 0.05 equiv. of palladium acetate, 0.1 equiv. of triphenylphosphine, 1 equiv. of tetrabutylammonium bromide and 2 equiv. of potassium carbonate in MeCN. Surprisingly, compared to our previous work on aryl bearing a β-iodo α,β-unsaturated carboxylic moiety, assays in dimethyl formamide ended-up with extremely poor yield. No evidence for the moment has been found for the moment to explain that observation. The use of a phosphine ligated palladium (0) catalyst such as tetrakis(triphenylphosphine)-palladium(0) is also suitable for the transformation, giving very similar yields. As expected, in the absence of Pd, no reaction occurred. The reaction requires temperatures of at least 80 °C to proceed. Below that temperature, no reaction was observed and starting materials were fully recovered. Compared to terminal alkynes used in Sonogashira like reactions, allenyltin offers a major advantage in terms of regioselectivity (see Scheme 3). Published experiments using a Pd catalyzed tandem Sonogashira/azacyclization or Ag catalyzed intramolecular Csp-azacyclization resulted in a mixture of 5-*exo dig* and 6-*endo dig* cyclization products [40,41]. This is because alkynes offer two attack areas resulting in two possible cyclization products (see Scheme 3, path a). Unlike the latter, the allene structure has a well-defined electrophilic area located on the digonal carbon (see Scheme 3, path b). This provides a regiospecific outcome to the reaction and therefore an undeniable advantage in terms of selectivity in comparison to alkyne cyclizations. In addition, allenyltin reagents offer an important feature as they can be used to transfer small volatile fragments such as C3 hydrocarbon because of the heavy weight of the trialkyltin group.

**Scheme 4:**
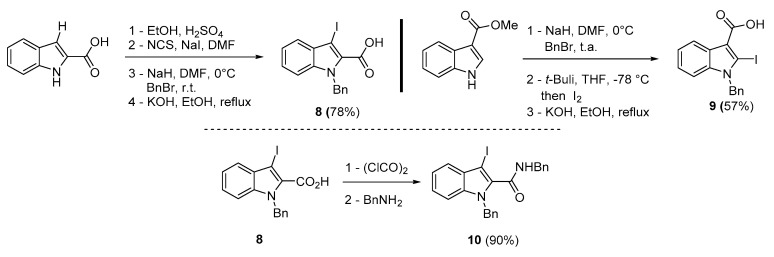
Synthesis of 3 (or 2)-iodoindole-2(or 3)-carboxylic acid **8**, **9** and 3-iodoindole-2-carboxamide **10**.

To broaden the scope of the use of allenyltin reagents, we extended our investigations to indole derivatives bearing a β-iodo α,β-unsaturated carboxylic or carboxamide moiety (**8, 9** and **10**). Compounds **8**, **9** and **10** were synthesized in good yields starting from the corresponding commercial indoles (Scheme 4). Compound **8** was obtained in four steps from (1*H*)indole-2-carboxylic acid [42,43]. After esterification of the starting indole and halogenation of the ester with *N*-chlorosuccinimide/sodium iodide (NCS/NaI) in DMF, the resulting indole was reacted with benzylbromide and saponified into **8** in good yield. It was impossible to obtain indole **9** in the same way. Methyl indole-3-carboxylate was benzylated prior to halogenation and saponification. A treatment with *t*-BuLi and molecular iodine led to the synthesis of **9** in good yield [42], while the use of *n*-BuLi or *s*-BuLi led to moderate to poor yields. Compound **10** was obtained in good yield by treatment of **8** with oxalyl chloride followed by a reaction with benzylamine [27]. Having the starting materials, we subjected them to react with allenyltin derivatives (Table 1). No significant difference in the behavior of the transformation between aromatic and heteroaromatic substrates was found, except for the fact that DMF proved to be surprisingly inefficient and led to poor yields.

As expected, **10** led to the synthesis of β-carbolinone **2** (entry 6), showing an efficient route to that important class of alkaloids. Note that in the case of amide **10**, the replacement of the benzyl group with the acetyl group proved to be ineffective, as only the formation of a few traces of cyclization product was observed, indicating that the nucleophilicity of the amine is an essential parameter in this heterocyclization reaction. In the same way, **8** and **9** afforded indolo[2,3-*c*]pyran-1-ones **3a**–**c** and indolo[3,2-*c*]pyran-1-ones **4a**,**b** respectively with reasonable to good yields. A certain number of methods to access these indolopyranones have been reported in the literature. For example, compounds **3** and **4** can be accessed from γ-ketoester cyclization [44], anhydride rearrangement [45,46], metal-catalyzed enyne cyclization [47,48], or metal-catalyzed coupling [49,50]. We also published recently a convenient Cu-catalyzed domino route to these type of molecules. However, the present study shows that although the copper catalyzed process is cheaper in term of catalyst, the tandem Stille coupling/heterocyclization using allenyltin reagents offers the possibility of accessing a wide variety of new heterocyclic compounds, and the reaction requires a lower temperature than in the case of Cu-catalyzed cyclization. Likewise, this strategy has been successfully extended to 3-iodothiophene-2-carboxylic acid and 3-iodoimidazo[1,2-*a*]pyridin-2-carboxylic acid to lead, with good yields, to thieno[2,3-*c*] pyran-7(7*H*)-one **5** and pyrano[3’,4’:4,5]imidazo[1,2-*a*]pyridin-1-one **6**, respectively (entries 12 and 13). Note that few synthesis of this type of compound has been reported to date [51,52].

## 3. Materials and Methods

### 3.1. General Protocol for Synthesis of 1,2-Benzothiazine 1,1-dioxides 1

To a Schlenk tube, under argon, containing 3.1 mmol of sulfonamide **6** in CH_3_CN (10 mL), potassium carbonate (860 mg, 2 equiv.), tetrabutylammonium bromide (1 g, 1 equiv.), triphenylphosphine (80 mg, 10 mol %) and Pd(OAc)_2_ (35 mg, 5 mol %) were added successively. The mixture was degassed, placed under nitrogen and well-stirred during 10 min. Allenyltributyltin (5.9 mmol, 1 equiv.) was then added. After 4 h at reflux, the mixture was hydrolyzed with water, and the organic phases were extracted with ethyl acetate. The combined organic extracts were dried over MgSO_4_, concentrated under vacuum, and the resulting residue was purified by silica gel column chromatography (petroleum ether/diethyl ether = 90/10) to afford the desired product **1**.

*2-Benzyl-3,7-dmethyl-1,2-benzothiazine 1,1-dioxide* (**1a**), 71%; yellow solid; mp: 119–121 °C; ^1^H NMR (300 MHz, CDCl_3_, ppm) *δ*: 7.83 (1H, d, *J* = 8.1 Hz), 7.30–7.22 (m, 4H), 7.14–7.12 (m, 3H), 6.13 (s, 1H), 5.05 (s, 2H), 2.47 (s, 3H), 2.17 (s, 3H). ^13^C NMR (75 MHz, CDCl_3_, ppm) *δ*: 142.8, 139.8, 137.1, 133.3, 129.1 (2C), 128.8, 128.3, 127.9, 127.0 (2C), 126.9, 121.9, 110.0, 48.7, 22.0, 21.2. LR-MS (EI, 70 eV): *m*/*z* (%): 299 [M]^+^ HR-MS (ESI): Anal. Calcd for C_17_H_17_NO_2_S [M + H]^+^ 300.0980, found 300.0995.

*2-Benzyl-7-methyl-3-pentyl-1,2-benzothiazine 1,1-dioxide* (**1b**), 80%; yellow solid; mp: 122–124 °C. ^1^H NMR (300 MHz, CDCl_3_, ppm) *δ*: 7.83 (d, *J* = 8.0 Hz, 1H). 7.30–7.03 (m, 7H_ar_), 6.23 (s, 1H), 4.99 (s, 2H), 2.46 (s, 3H), 2.38 (t, *J* = 7.7 Hz, 2H), 1.67–1.25 (m, 6H), 0.94 (t, *J* = 6.7 Hz, 3H). ^13^C NMR (75 MHz, CDCl_3_, ppm) *δ*: 143.8, 142.8, 137.1, 133.2, 129.0 (2C), 128.6, 128.5, 127.9, 127.3, 127.2 (2C), 121.8, 111.0, 49.2, 33.9, 31.2, 27.1, 22.0, 21.2, 14.2,. HRMS (ESI) *m*/*z* calcd for C_21_H_25_NO_2_S [M + H]^+^ 356.1606, found 356.1622.

*2-Benzyl-3-ethyl-7-methyl-1.2-benzothiazine 1,1-dioxide* (**1c**), 77%; orange solid; mp: 119–121 °C. ^1^H NMR (300 MHz, CDCl_3_, ppm) *δ*: 7.81 (d, *J* = 8.0 Hz, 1H), 7.31–7.05 (m, 7H_ar_), 6.24 (s, 1H), 5.01 (s, 2H), 2.47 (s, 3H), 2.42 (q, *J* = 7.3 Hz, 2H), 1.23 (t, *J* = 7.3 Hz, 3H). ^13^C NMR (75 MHz, CDCl_3_, ppm) *δ*: 144.6, 142.3, 136.7, 132.8, 129.1, 128.5 (2C), 128.1, 127.4, 126.9, 126.7 (2C), 121.5, 109.3, 48.5, 26.5, 21.6, 11.7. HRMS (ESI) *m*/*z* calcd for C_18_H_19_NO_2_S [M + H]^+^ 314.1215, found 314.1205.

*3,7-Dimethyl-2-(1-Phenyl-ethyl)-1.2-benzothiazine 1,1-dioxide* (**1d**), 76% yield, white solid; mp: 160–162 °C. ^1^H NMR (300 MHz, CDCl_3_, ppm) *δ*: 7.81 (d, *J* = 8.0 Hz, 1H), 7.33–7.28 (m, 6H_ar_), 7.12 (s, 1H), 6.27 (s, 1H), 5.68 (q, *J* = 7.1 Hz, 1H), 2.47 (s, 3H), 1.88 (s, 3H), 1.68 (d, *J* = 7.1 Hz, 3H), ^13^C NMR (75 MHz, CDCl_3_, ppm) *δ*: 142.4, 140.7, 139.7, 132.9, 129.6, 128.4, 128.2 (2C), 127.3 (2C), 126.7, 126.6, 121.6, 113.8, 55.7, 22.5, 21.6, 19.4. HRMS (ESI) *m*/*z* calcd for C_18_H_20_NO_2_S [M + H]^+^ 314.1215, found 314.1211.

*2-Allyl-3,7-dimethyl-1.2-benzothiazine 1,1-dioxide* (**1e**), 73%; yellow oil; ^1^H NMR (300 MHz, CDCl_3_, ppm) *δ*: 7.83 (d, *J* = 8.1 Hz, 1H), 7.30-7.24 (m, 1H), 7.13 (s, 1H), 6.21 (s, 1H), 5.88–5.69 (m, 1H), 5.17–5.12 (m, 2H), 4.34 (dt, *J* = 5.0 Hz, *J* = 1.6 Hz, 2H), 2.46 (s, 3H), 2.26 (s, 3H). ^13^C NMR (75 MHz, CDCl_3_, ppm) *δ*: 142.2, 139.3, 133.0, 132.7, 128.1, 127.7, 126.2, 121.2, 117.1, 109.1, 46.9, 21.4, 20.3. HRMS (ESI) *m*/*z* calcd for C_13_H_16_NO_2_S [M + H]^+^ 250.0896, found 250.0895.

### 3.2. General Protocol for Synthesis of 2,9-Dihydro-1H-pyrido[3,4-b]indol-1-one Derivated **2**–**6**

Potassium carbonate (860 mg, 2 equiv.), tetrabutylammonium bromide (1 g, 1 equiv.), triphenylphosphine (80 mg, 10 mol %) and Pd(OAc)_2_ (35 mg, 5 mol %) were added successively to a Schlenk tube, under argon, containing the indole derivative **6**, **7** or **8** (3.1 mmol) in CH_3_CN (10 mL). The mixture was degassed, placed under nitrogen and well-stirred during 10 min. Allenyltributyltin (5.9 mmol, 1 equiv.) was then added. After 8h at reflux, the mixture was hydrolyzed with water and the organic phases were extracted with ethyl acetate. The combined organic extracts were dried over MgSO_4_, concentrated under vacuum and the resulting residue was purified by silica gel column chromatography (petroleum ether/diethyl ether = 80/20) to afford the desired product.

*2,9-Dibenzyl-3-methyl-2,9-dihydro-1H-pyrido[3,4-b]indol-1-one* (**2**), 73%; beige solid; mp: 119–121 °C. ^1^H NMR (300 MHz, CDCl_3_, ppm) *δ*: 7.95 (d, *J* = 7.8 Hz, 1H), 7.44 (d, *J* = 6.0 Hz, 2H), 7.30–7.11 (m, 11H), 6.83 (s, 1H), 6.13 (s, 2H), 5.51 (s, 2H), 2.43 (s, 3H). ^13^C NMR (75 MHz, CDCl_3_, ppm) *δ*: 156.7, 140.3, 138.2, 136.9, 135.8, 129.1, 128.3 (2C), 128.0 (2C), 126.6 (2C), 126.4 (2C), 125.7, 125.1, 124.5, 121.1, 120.7, 119.5, 100.5, 100.2, 47.3, 46.3, 20.5. (^1^H NMR and ^13^C NMR of compounds **1a**–**e**, **2**, **3a**–**c**, **4a**,**b** are in Supplementary Materials). HRMS (ESI) *m*/*z* calcd for C_26_H_23_N_2_O [M + H]^+^ 379.1805, found 379.1804.

*9-Benzyl-3-butylpyrano[3,4-b]indol-1(9H)-one* (**3a**), 56%; orange solid; mp: 90–91 °C; ^1^H NMR (300 MHz, CDCl_3_, ppm) *δ*: 7.86 (d, *J =* 8.0 Hz, 1H), 7.48–7.41 (m, 2H), 7.31–7.10 (m, 6H), 6.72 (s, 1H), 5.93 (s, 2H), 2.65 (t, *J =* 7.5 Hz, 2H), 1.77–1.73 (m, 2H), 1.46–1.42 (m, 2H), 0.98 (t, *J =* 7.4 Hz, 3H). ^13^C NMR (75 MHz, CDCl_3_, ppm) *δ*: 157.8, 157.5, 141.0, 137.7, 128.7, 128.0, 127.6, 127.2, 126.5, 121.7, 121.3, 120.9, 120.2, 111.4, 98.2, 48.0, 33.4, 29.6, 22.3, 13.9. HRMS (ESI) *m*/*z* calcd for C_22_H_21_NO_2_ [M + H]^+^ 332.1645, found 332.1650.

*9-Benzyl-3-hexylpyrano[3,4-b]indol-1(9H)-one* (**3b**), 62%; yellow oil; ^1^H NMR (300 MHz, CDCl_3_, ppm) *δ*: 7.90 (d, *J* = 8.0 Hz, 1 H), 7.48–7.46 (m, 2 H), 7.31–7.23 (m, 6 H), 6.72 (s, 1 H), 5.94 (s, 2 H), 2.65 (t, *J* = 7.5 Hz, 2 H), 1.78–1.76 (m, 2 H), 1.44–1.34 (m, 6 H), 0.92 (t, *J* = 6.9 Hz, 3 H). ^13^C NMR (75 MHz, CDCl_3_, ppm) *δ*: 157.7, 157.4, 140.9, 137.6, 128.6 (2 C), 127.9, 127.5, 127.1 (2 C), 126.4, 121.6, 121.2, 120.8, 120.1, 111.3, 98.0, 47.9, 33.6, 31.6, 28.8, 27.4, 22.6, 14.1. HRMS (ESI) *m*/*z* calcd for C_24_H_26_NO_2_ [M + H]^+^ 360.1885, found 360.1889.

*3-Hexyl-9-methylpyrano[3,4-b]indol-1(9H)-one* (**3c**), 76%; white solid; mp: 88–90 °C; ^1^H NMR (300 MHz, CDCl_3_, ppm) *δ*: 7.88 (d, *J* = 8.1 Hz, 1 H), 7.55 (dd, *J* = 8.5, 6.8, 1.1 Hz, 1 H), 7.47 (d, *J* = 8.5 Hz, 1 H), 7.27 (dd, *J* = 8.1, 6.8, 1.1 Hz, 1 H), 6.70 (s, 1 H), 4.22 (s, 3 H), 2.64 (t, *J* = 7.5 Hz, 2 H), 1.76 (qt, *J* = 7.5 Hz, 2 H), 1.42–1.27 (m, 6 H), 0.91 (t, *J* = 7.0 Hz, 3 H). ^13^C NMR (75 MHz, CDCl_3_, ppm) *δ*: 157.9, 157.0, 141.3, 127.7, 125.8, 121.6, 120.8, 120.6 (2 C), 110.5, 98.1, 33.6, 31.6, 31.2, 28.7, 27.5, 22.5, 14.0. HRMS (ESI) *m*/*z* calcd for C_18_H_22_NO_2_ [M + H]^+^ 284.1572, found 284.1576.

*5-Benzyl-3-methylpyrano[4,3-b]indol-1(5H)-one* (**4a**), 57%; yellow solid; mp: 115–117 °C; ^1^H NMR (300 MHz, CDCl_3_, ppm) *δ:* 8.28–8.21 (m, 1H), 7.38-7.22 (m, 6H), 7.25–7.23 (m, 2H), 6.33 (q, *J* = 0.8 Hz, 1H), 5.42 (s, 2H), 2.39 (d, *J* = 0.8 Hz, 3H). ^13^C NMR (75 MHz, CDCl_3_, ppm) *δ*: 160.6, 160.1, 146.7, 138.4, 135.7, 129.2 (2C), 128.2, 126.3 (2C), 124.6, 124.5, 122.9, 121.4, 109.9, 99.5, 93.5, 47.3, 20.8. HRMS (ESI) *m*/*z* calcd for C_19_H_16_NO_2_ [M + H]^+^ 290.1175, found 290.1173.

*5-Benzyl-3-pentylpyrano[4,3-b]indol-1(5H)-one* (**4b**), 60%; colorless solid; mp: 123–125 °C. ^1^H NMR (300 MHz, CDCl_3_, ppm) *δ*: 8.03–7.97 (m, 1H), 7.35–7.27 (m, 6H), 7.12–7.03 (m, 3H), 6.29 (s, 1H), 5.38 (s, 2H), 2.57 (t, *J* = 7.9 Hz, 2H,), 1.80–1.65 (m, 2H), 1.35–1.27 (m, 4H), 0.88 (t, *J* = 6.4 Hz, 3H,). ^13^C NMR (75 MHz, CDCl_3_, ppm) *δ*: 164.5, 160.2, 146.7, 138.4, 135.7, 129.2 (2C), 128.2, 126.3 (2C), 124.5, 122.8, 122.2, 121.4, 109.9, 99.6, 92.7, 47.7, 34.6, 31.3, 27.1, 22.5, 14.1. HRMS (ESI) *m*/*z* calcd for C_23_H_23_NO_2_ [M + H]^+^ 346.1729, found 346.1736.

*5-Pentyl-7H-thieno[2,3-c]pyran-7(7H)-one* (**5**) [42], 72%, Yellow gum. The data of the spectroscopic analyzes (^1^H NMR and ^13^C NMR) of product **5** are in agreement with those described in the literature [42].

*3-Hexyl pyrano[3’,4’:4,5] imidazo[1,2-a]pyridin-1-one* (**6**) [53], 68%, Yellow solid, mp: 142–144 °C. The data of the spectroscopic analyzes (^1H^ NMR and ^13C^ NMR) of product **6** are in agreement with those described in the literature [53].

## 4. Conclusion

In summary, we have developed a general and convenient one step regioselective route for the preparation of 1,1-dioxide 1,2-benzothiazines*,* β-carbolinones and pyranoindoles via Stille coupling of aromatic or heteroaromatic halide derivatives and allenyltributyltins reagents. The transformation proceeded selectively and provided good to excellent yields of a variety of potentially bioactive activities of the targeted cores. The results obtained may lead to the use of allenyltin reagent as an excellent alternative to previously published methodologies for the scientists involved in the field.

## Figures and Tables

**Table 1:**
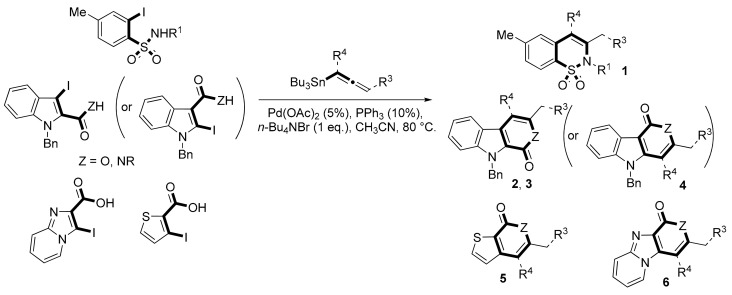
Synthesis of **1**, **2**, **3, 4, 5** and **6** via tandem Stille coupling/heterocyclization reaction.

Entry	R^1^	Z	Allenylstannane	Product	N°	Yield (%) ^a^
1	Bn		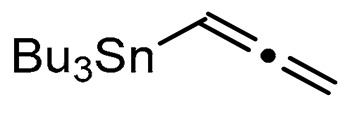	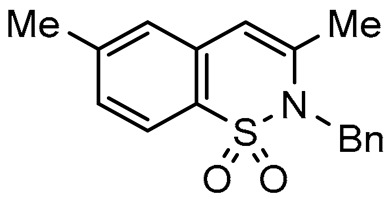	**1a**	71
2	Bn		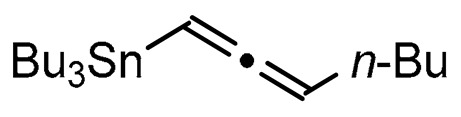	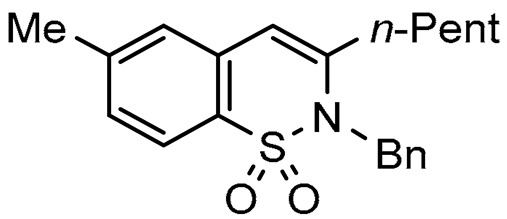	**1b**	80
3	Bn		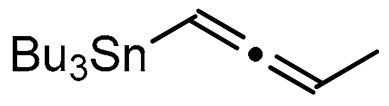	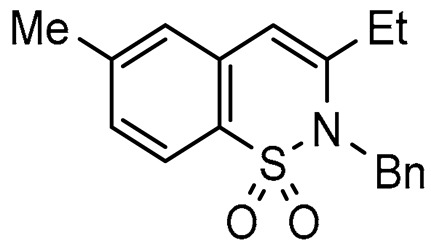	**1c**	77
4	CH(CH_3_)Ph		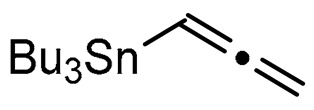	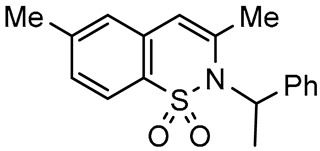	**1d**	76
5	Allyl		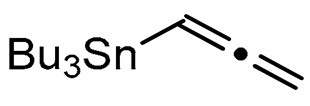	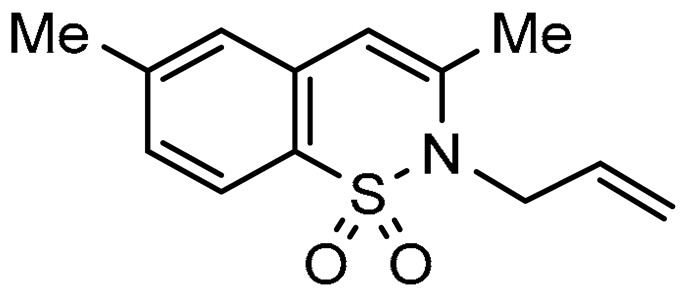	**1e**	73
6		*N*-Bn	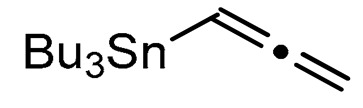	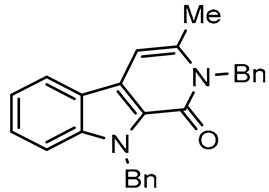	**2**	73
7		O	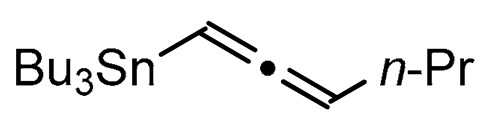	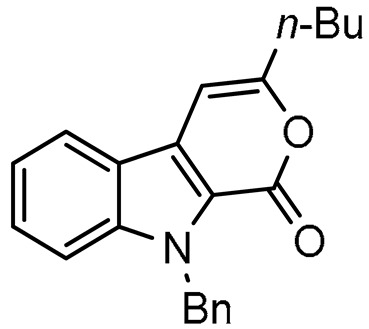	**3a**	56
8		O	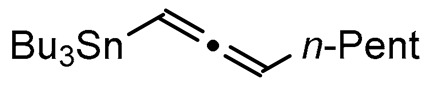	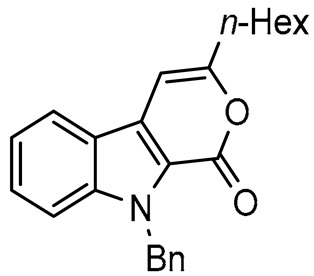	**3b**	62
9		O	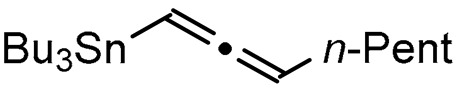	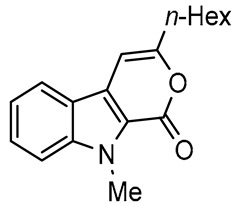	**3c**	76
10		O	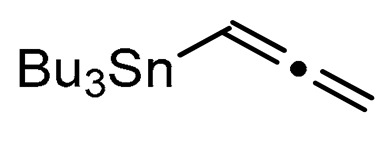	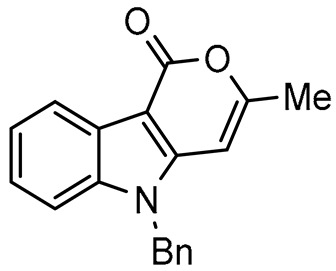	**4a**	57
11		O	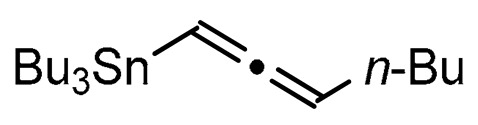	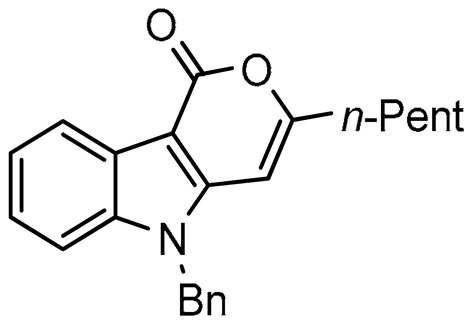	**4b**	60
12		O	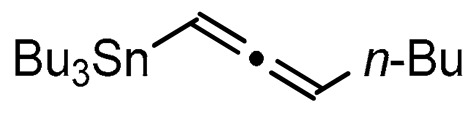	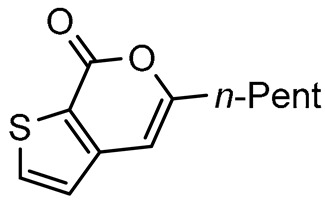	**5**	72
13		O	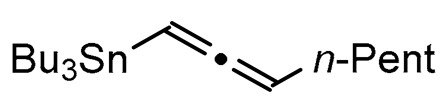	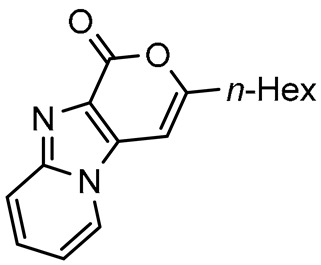	**6**	68

^a^ Isolated yield.

## Supplementary Materials

The following are available online. ^1^H-NMR and ^13^C-NMR of compounds **1a**–**e**, **2, 3a**–**c, 4a,b.**
