# Supplementary material for: A Direct and an Efficient Regioselective Synthesis of 1,2-Benzothiazine 1,1-dioxides, β-Carbolinones, Indolo[2,3-c]pyran-1-ones, Indolo[3,2-c]pyran-1-ones, Thieno[2,3-c]pyran-7-ones and Pyrano[3’,4’:4,5]imidazo[1,2-a]pyridin-1-ones via Tandem Stille/Heterocyclization Reaction
Source: Molecules. 2020 Nov 4;25(21):5137. doi: 10.3390/molecules25215137 (PMC7663652; doi:10.3390/molecules25215137)

molecules

Article

A Direct and an Efficient Regioselective Synthesis of 1,2-Benzothiazine 1,1-dioxides, *β*-carbolinones, Indolo[2,3-*c*]pyran-1-ones, Indolo[3,2-*c*]pyran-1-ones, Thieno[2,3-*c*]pyran-7-ones and Pyrano[3′,4′:4,5]imidazo[1,2-*a*]pyridin-1-ones Via Tandem Stille/Heterocyclization Reaction

Badr Jismy,[1] Khalil Cherry,[2] Carine Maaliki,[1] Samuel Inack-Ngi[4,] and Mohamed Abarbri[1,*]

[1] Laboratoire de Physico-Chimie des Matériaux et des Electrolytes pour l'Energie (PCM2E), EA 6299, Avenue Monge, Faculté des Sciences, Université de Tours, Parc de Grandmont, 37200 Tours, France;
[2] Laboratoire Matériaux, Catalyse, Environnement et Méthodes Analytiques (MCEMA), Campus Universitaire de Hadat, Université Libanaise.
[3] IKAMBA Organics, 39 rue des Granges Galands, 37550 Saint-Avertin, France.
* Correspondence: mohamed.abarbri@univ-tours.fr ; Tel.: +33(2)47-36-73-59; Fax: +33(2)47-36-70-73

*2-Benzyl-3-methyl-7-methyl-1,2-benzothiazine-1,1-dioxide(**1a**)*

^{1}H NMR (300 MHz, $CDCl_3$)

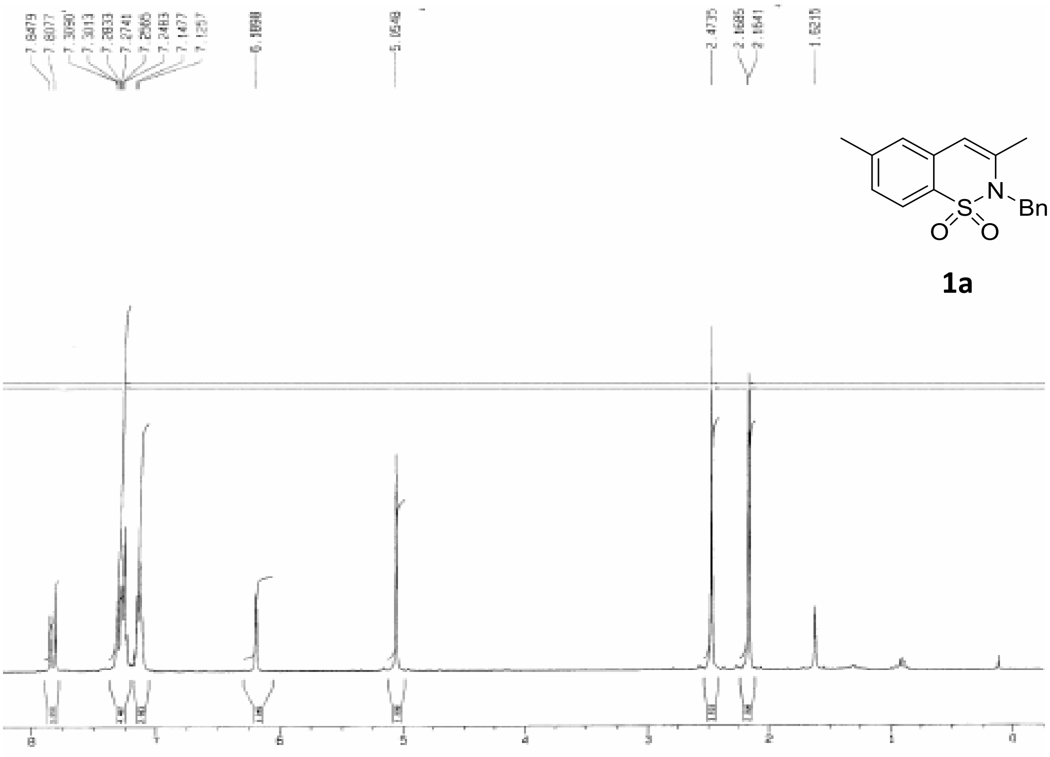

^{13}C NMR (75 MHz, $CDCl_3$)

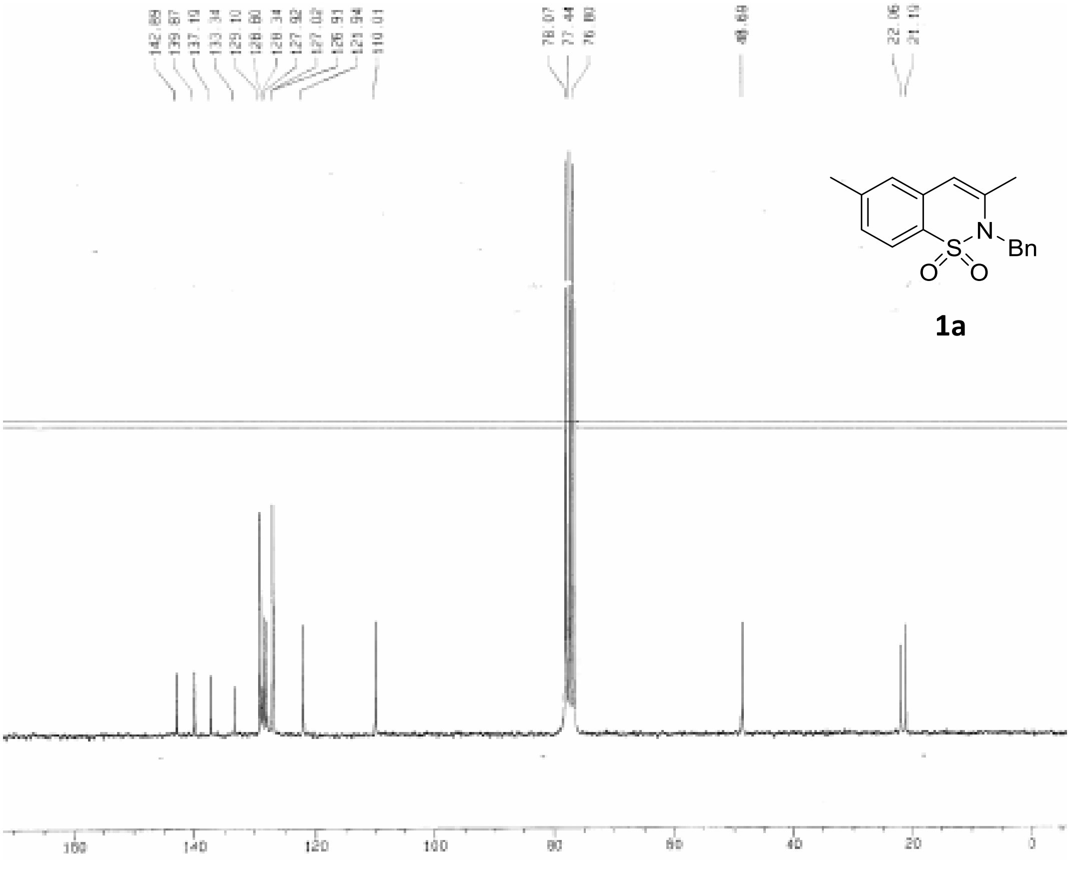

*2-Benzyl-3-pentyl-7-methyl-1,2-benzothiazine -1,1-dioxide (**1b**)*

^1H NMR (300 MHz, $CDCl_3$)

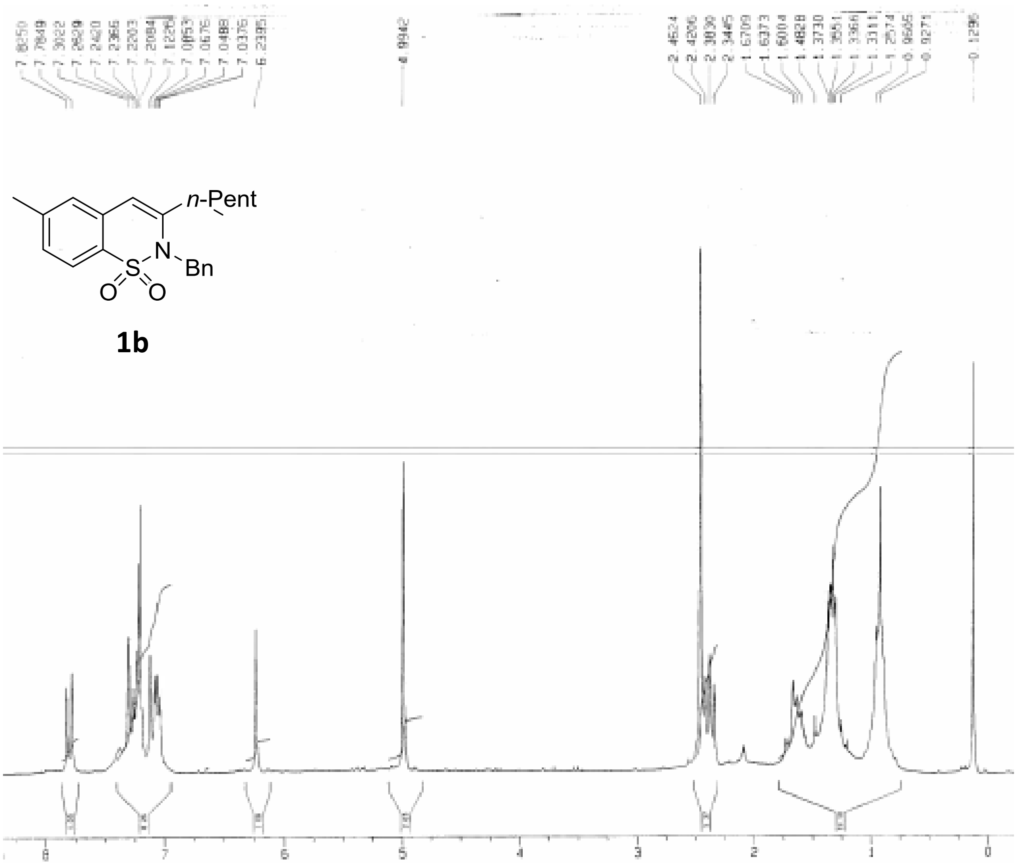

^{13}C NMR (75 MHz, $CDCl_3$)

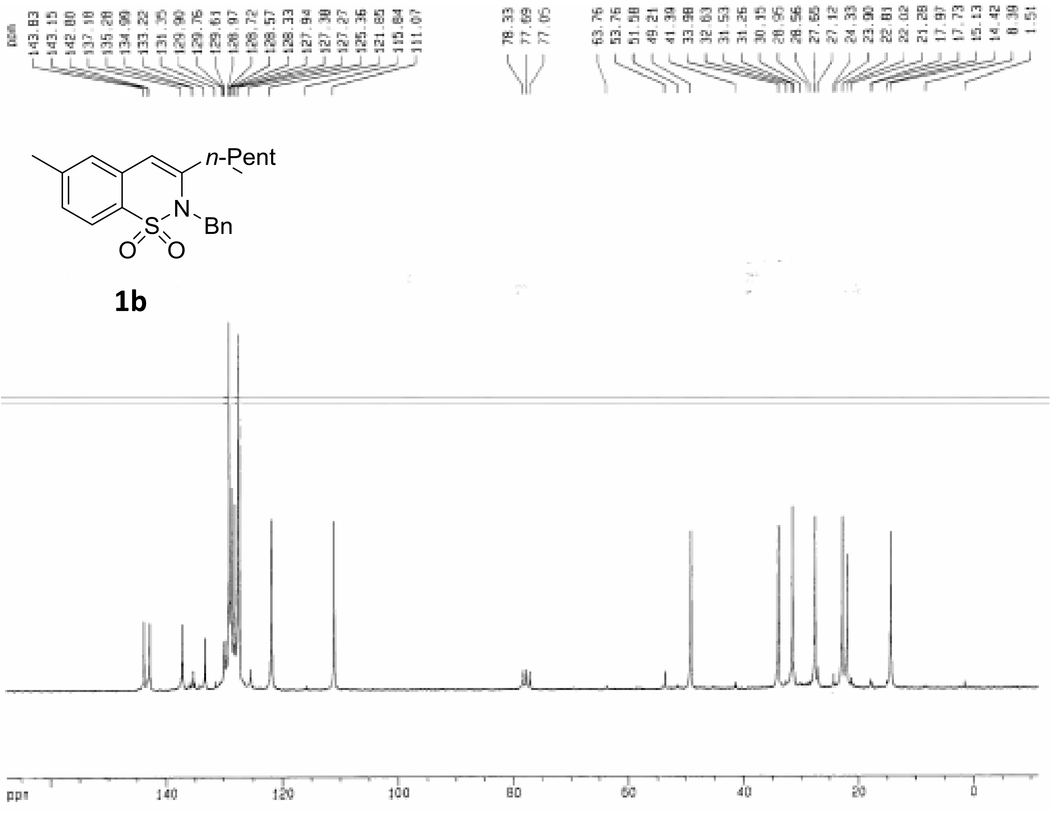

2-Benzyl-3-ethyl-7-methyl-1.2-benzothiazine-1,1-dioxide **(1c)**

^{1}H NMR (300 MHz, $CDCl_3$)

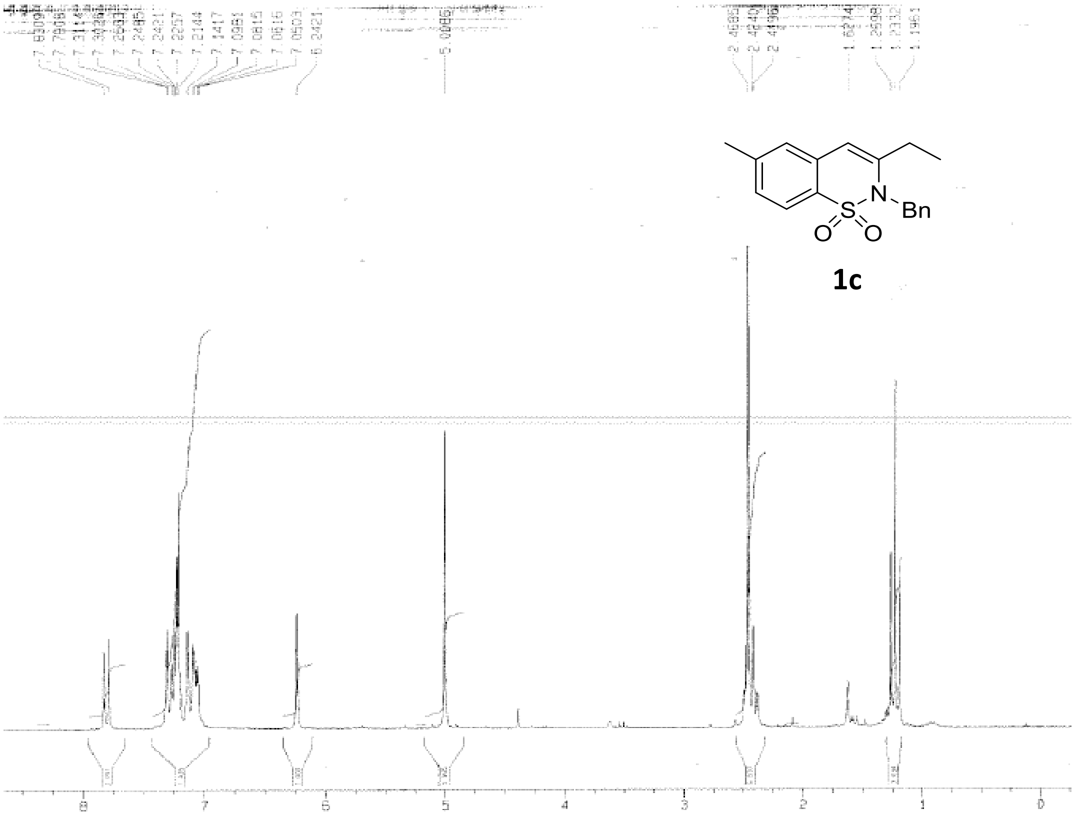

^{13}C NMR (75 MHz, $CDCl_3$)

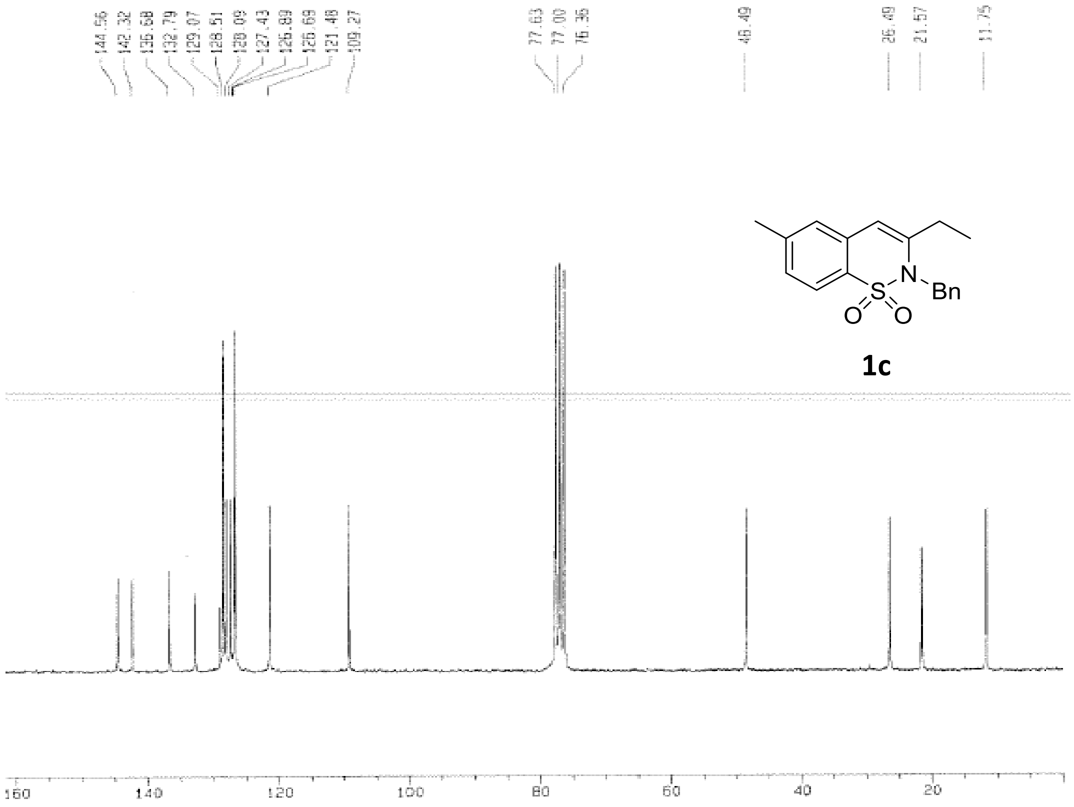

2-(1-Phenyl-ethyl)-3-methyl-7-methyl-1.2-benzothiazine-1,1-dioxide **(1d)**

^{1}H NMR (300 MHz, $CDCl_3$)

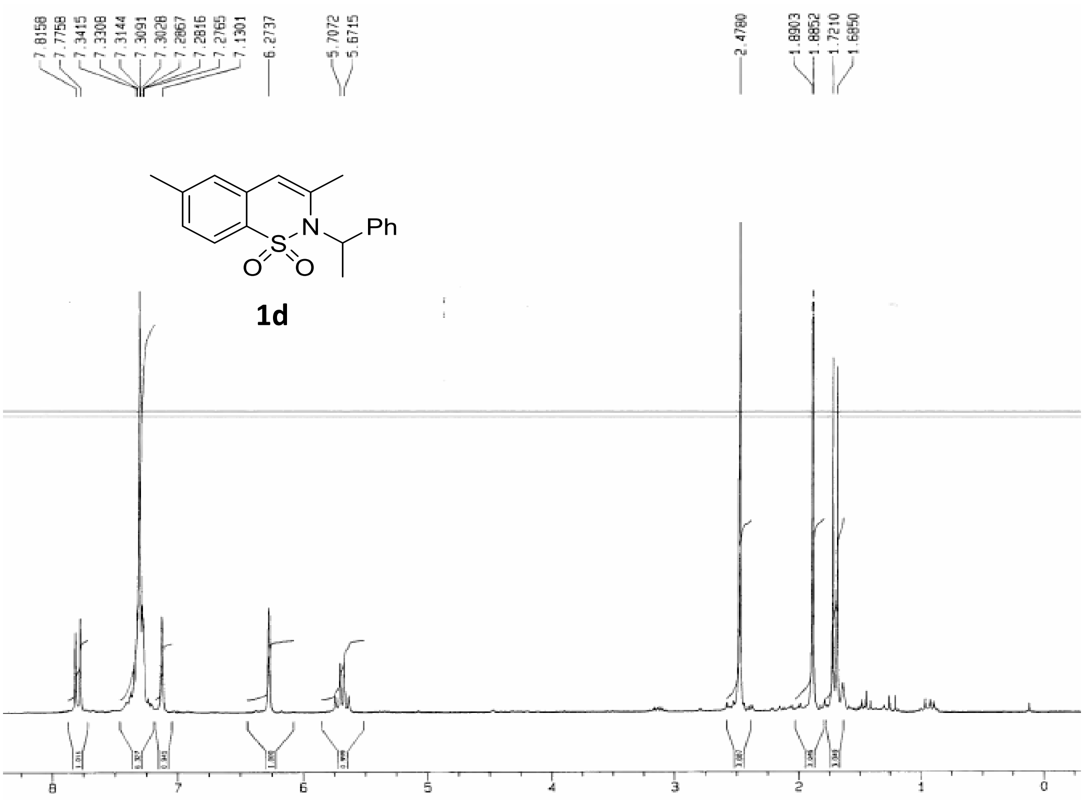

^{13}C NMR (75 MHz, $CDCl_3$)

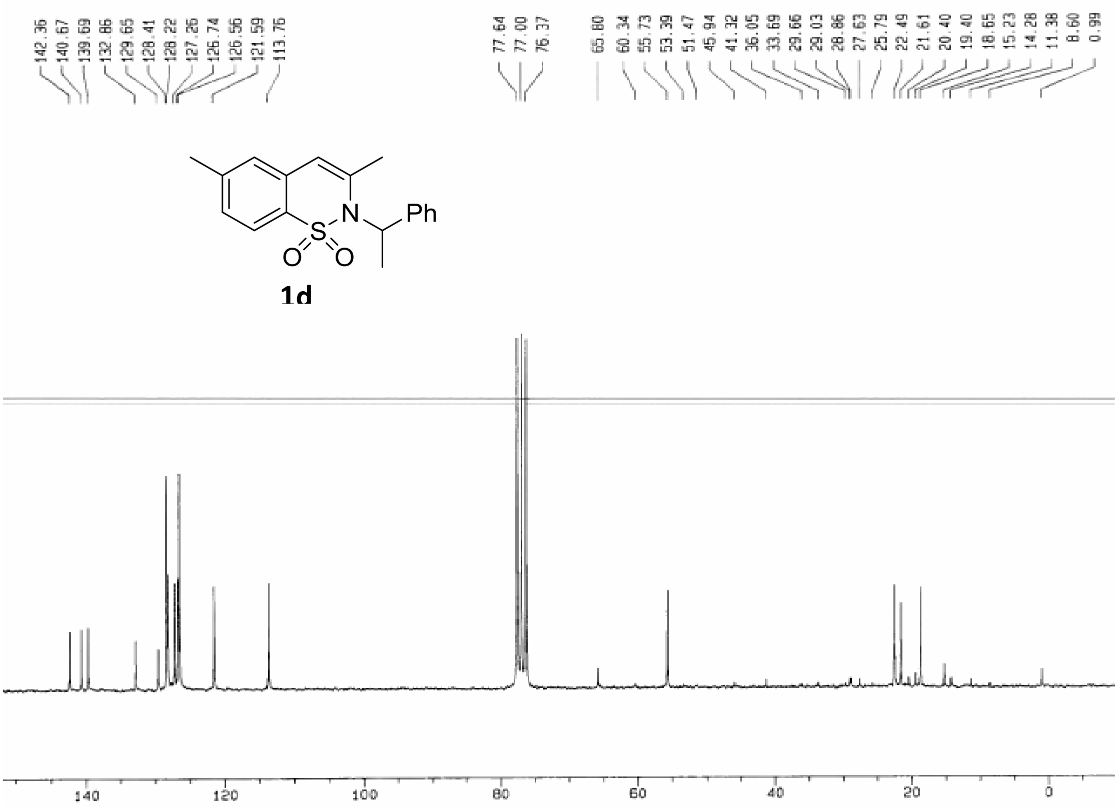

2-Allyl-3-methyl-7-methyl-1.2-benzothiazine-1,1-dioxide **(1e)**

^{1}H NMR (300 MHz, $CDCl_3$)

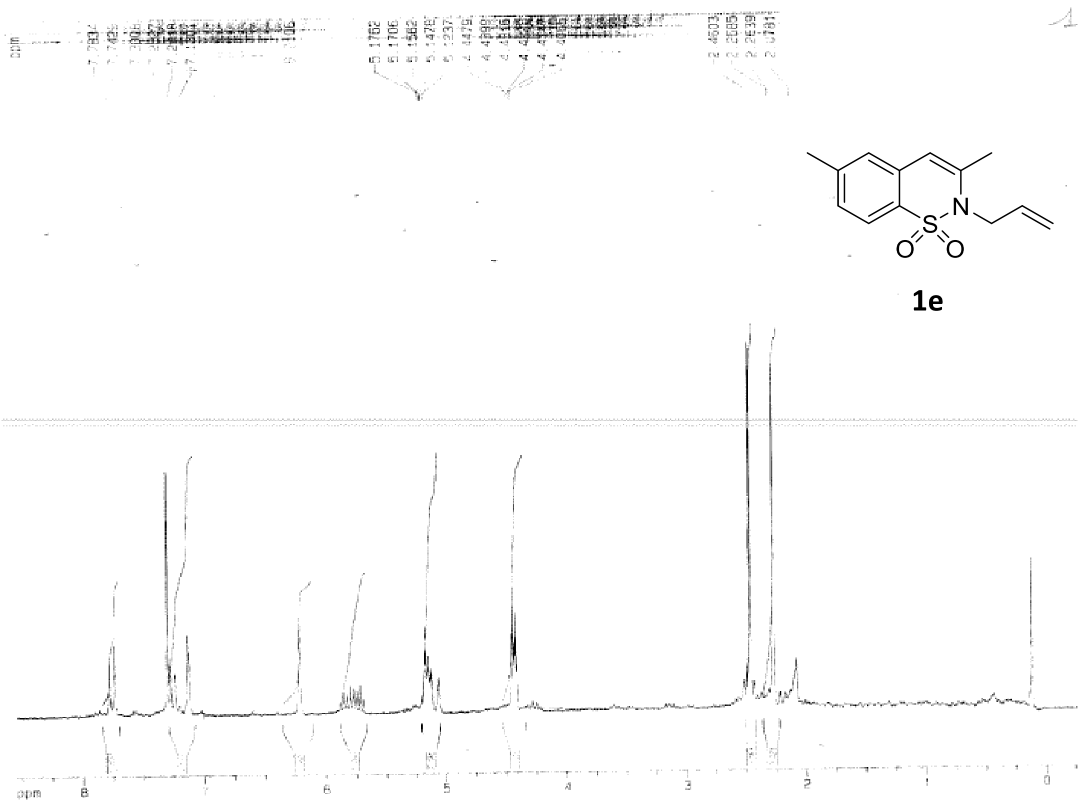

^{13}C NMR (75 MHz, $CDCl_3$)

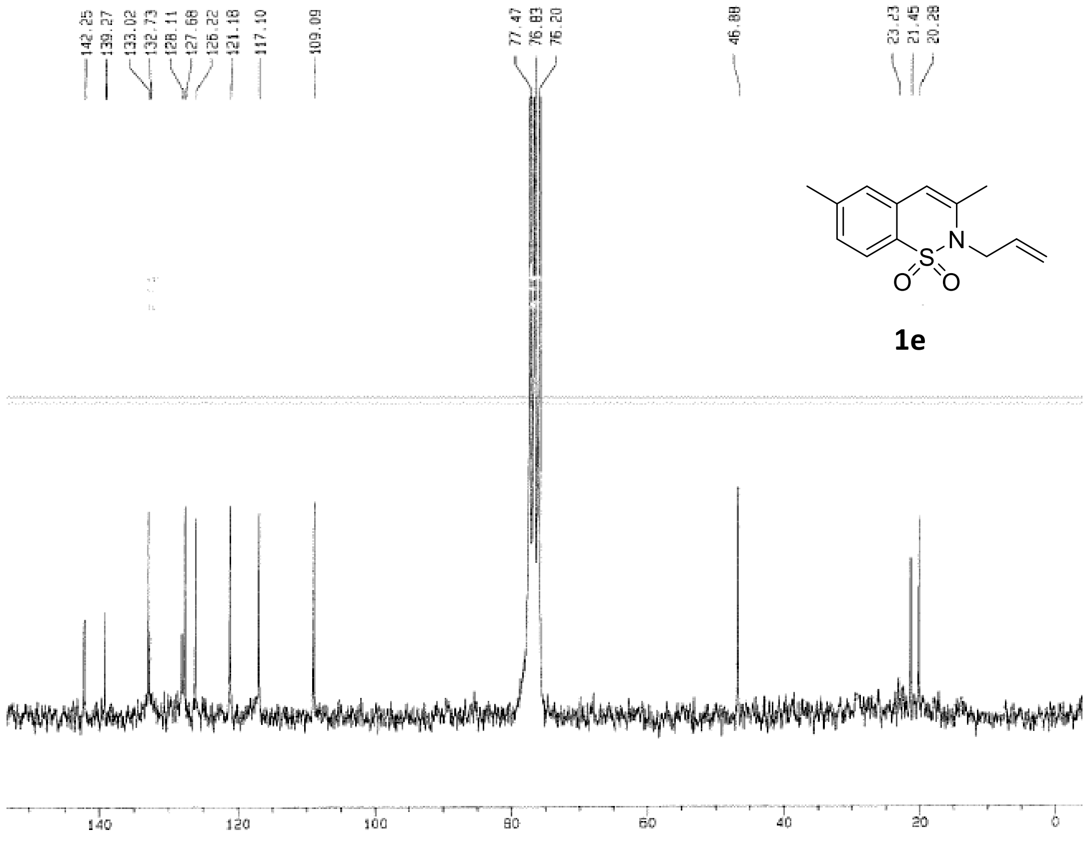

9-Benzyl-3-butylpyrano[3,4-b]indol-1(9H)-one ***(3a)***

^{1}H NMR (300 MHz, $CDCl_3$)

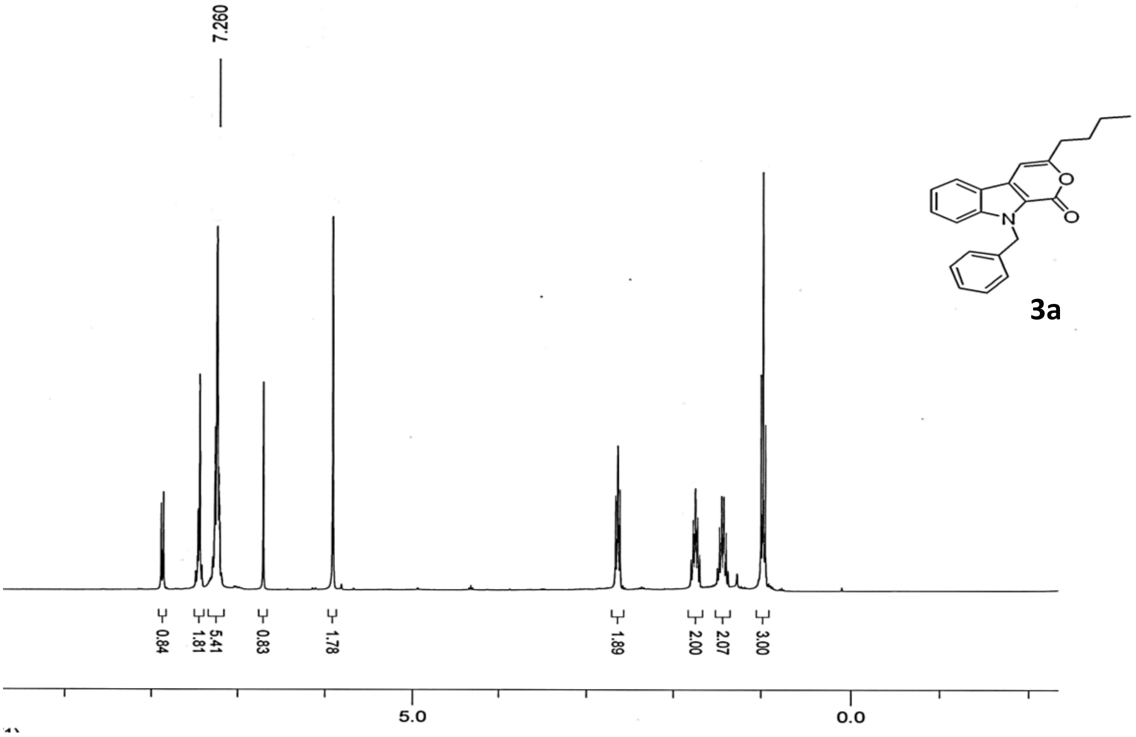

^{13}C NMR (75 MHz, $CDCl_3$)

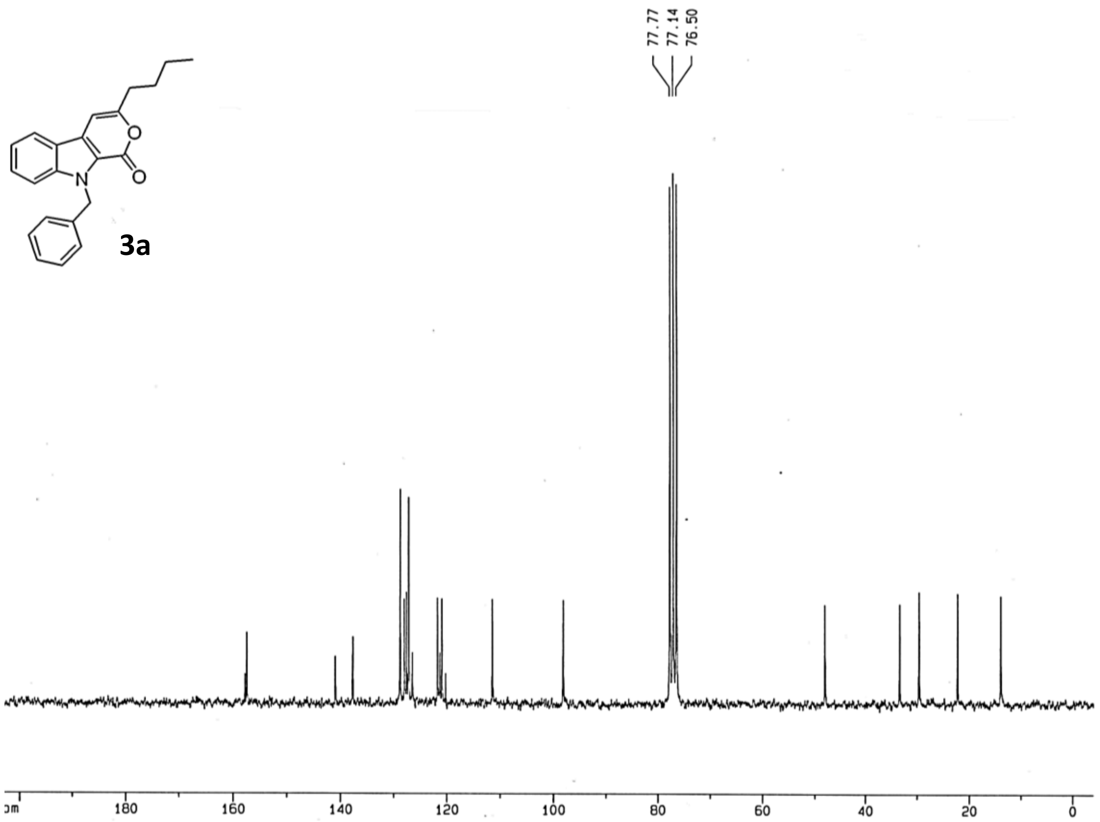

*9-benzyl-3-hexylpyrano[3,4-b]indol-1(9H)-one (**3b**)*

^{1}H NMR (300 MHz, $CDCl_3$)

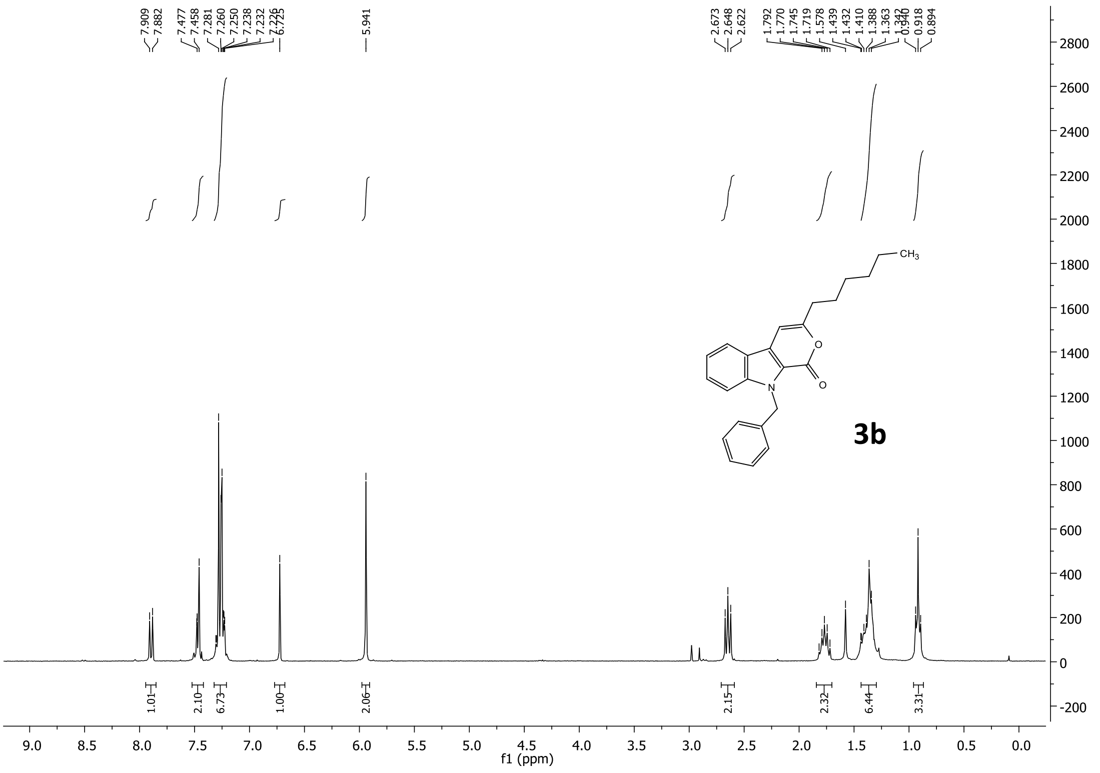

^{13}C NMR (75 MHz, $CDCl_3$)

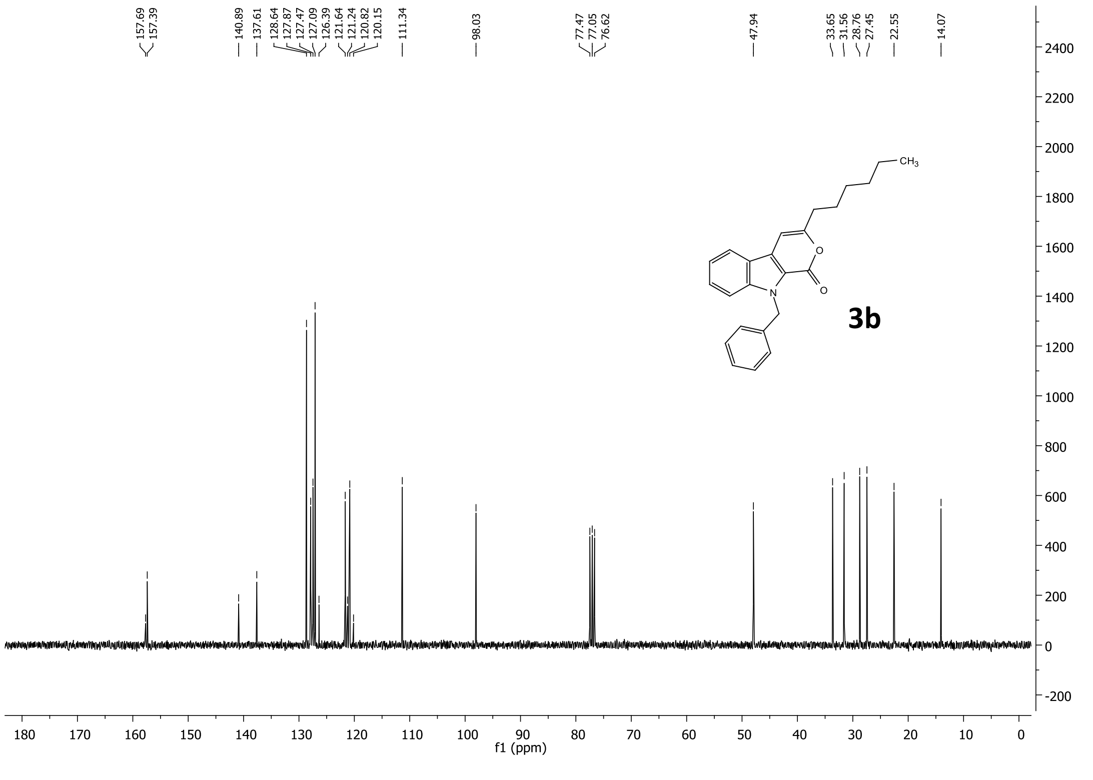

3-hexyl-9-methylpyrano[3,4-b]indol-1(9H)-one ***(3c)***

^{1}H NMR (300 MHz, $CDCl_3$)

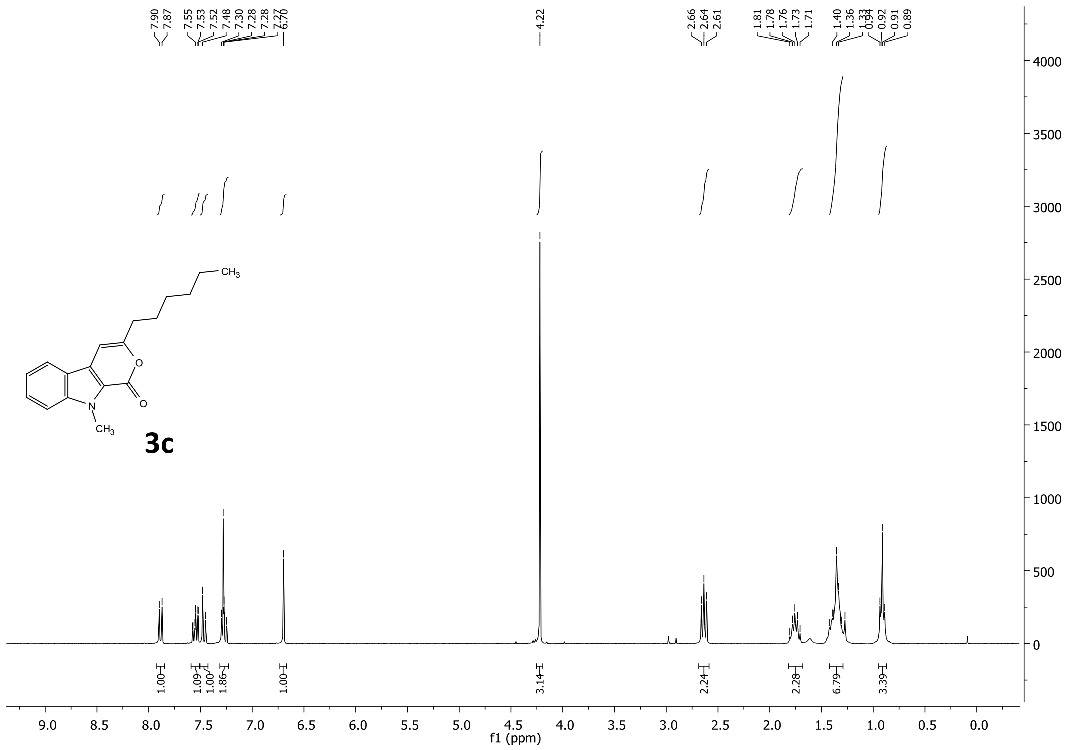

^{13}C NMR (75 MHz, $CDCl_3$)

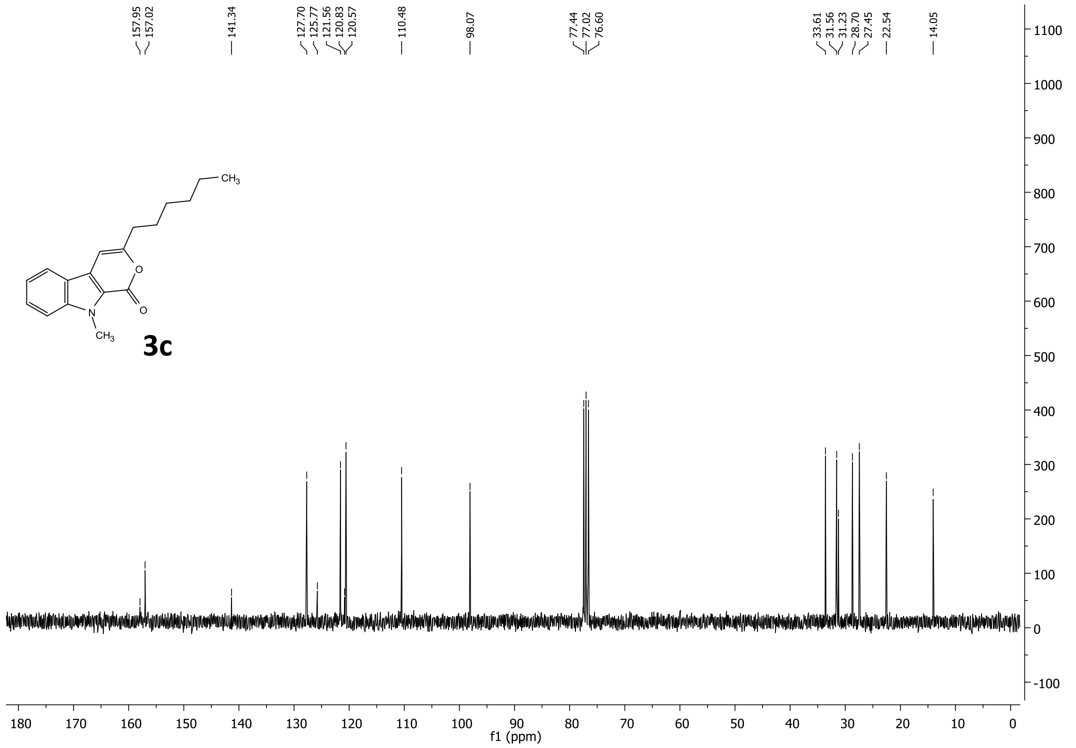

5-Benzyl-3-methylpyrano[4,3-b]indol-1(5H)-one ***(4a)***

^{1}H NMR (300 MHz, $CDCl_3$)

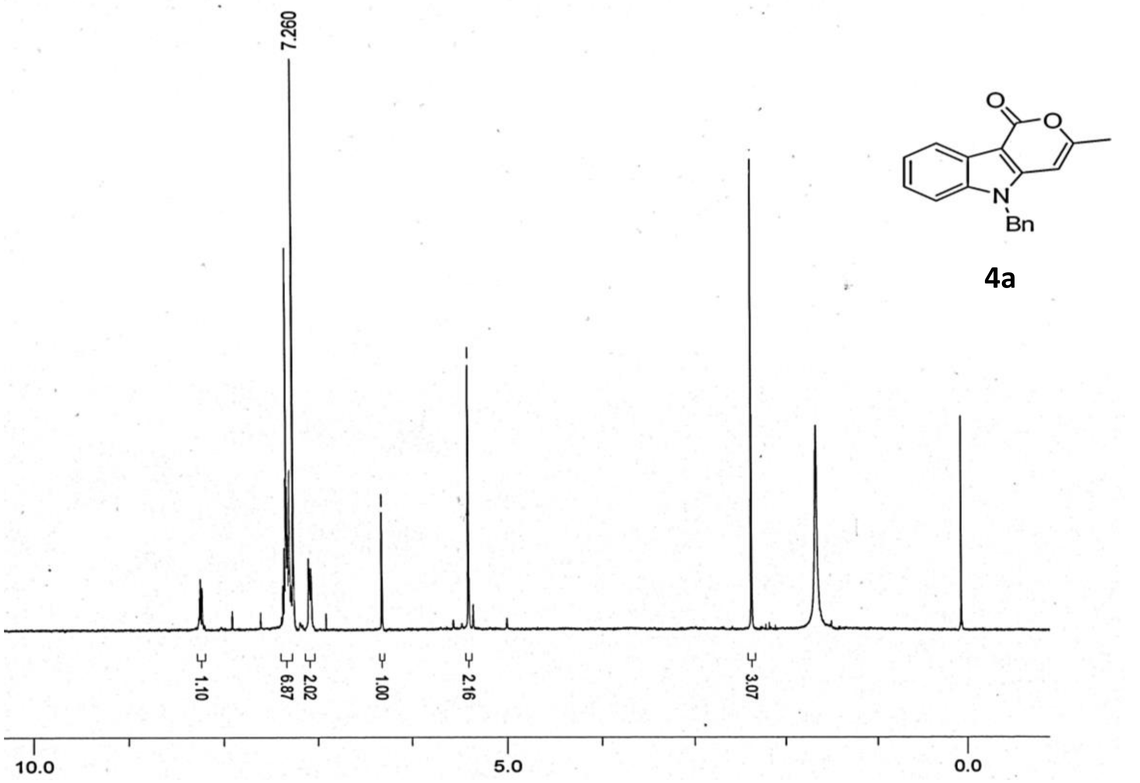

^{13}C NMR (75 MHz, $CDCl_3$)

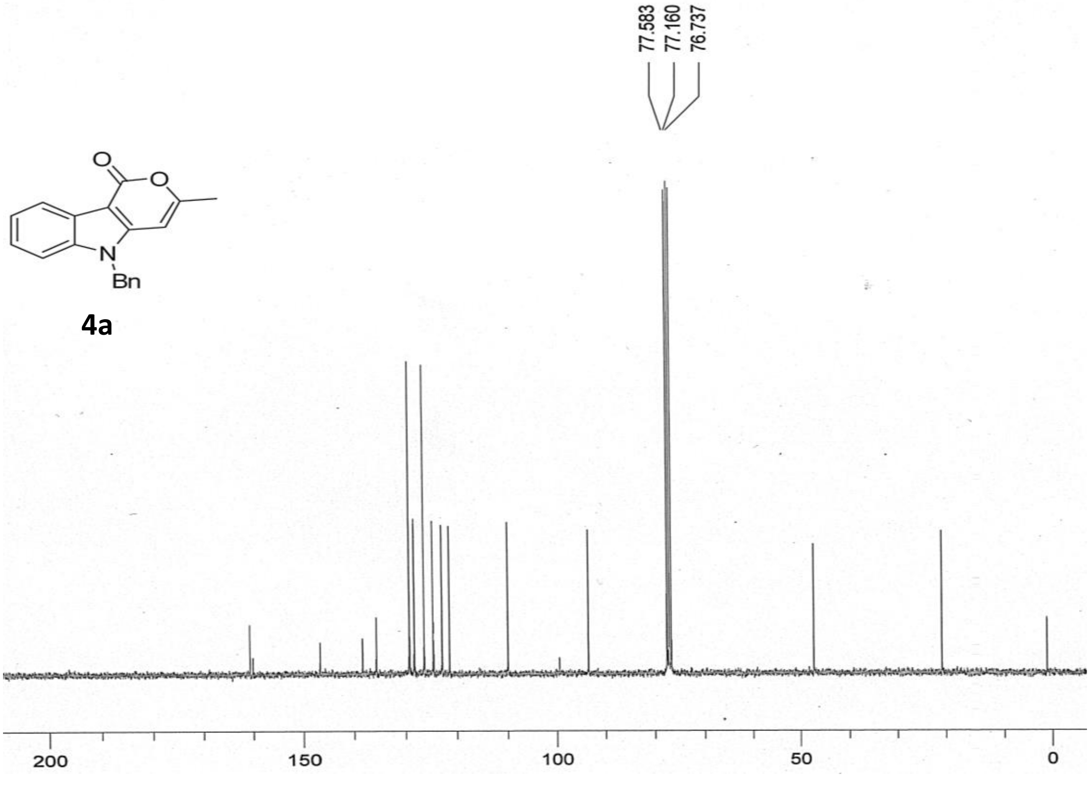

5-Benzyl-3-pentylpyrano[4,3-b]indol-1(5H)-one ***(4b)***

^{1}H NMR (300 MHz, $CDCl_3$)

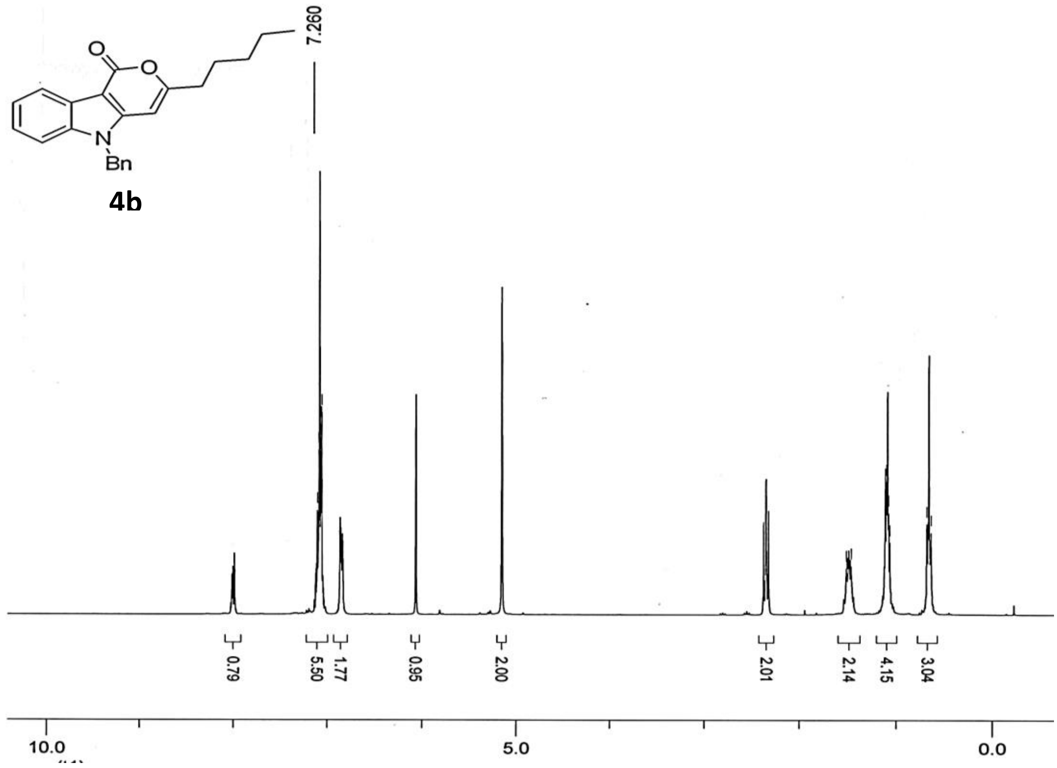

^{13}C NMR (75 MHz, $CDCl_3$)

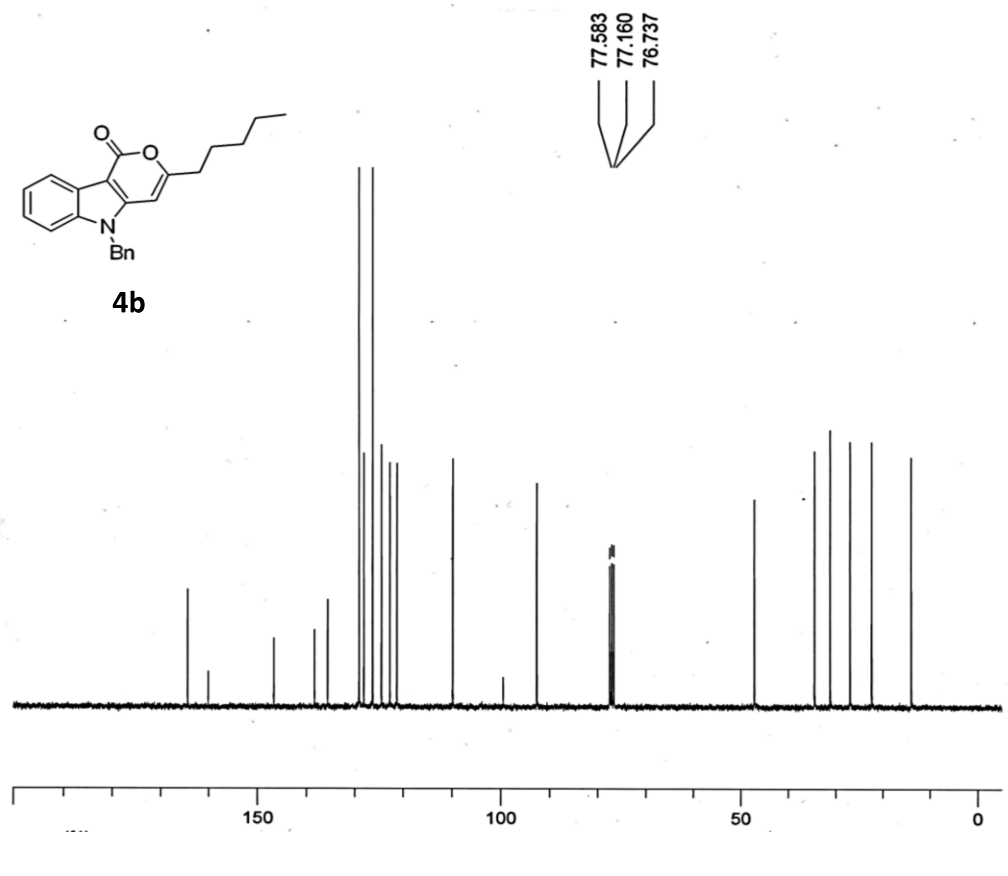

Supplement: Supplementary file 1 [file molecules-25-05137-s001.pdf]
